# Developing a nomogram for predicting depression in diabetic patients after COVID-19 using machine learning

**DOI:** 10.3389/fpubh.2023.1150818

**Published:** 2023-07-18

**Authors:** Haewon Byeon

**Affiliations:** ^1^Department of Digital Anti-aging Healthcare (BK21), Graduate School of Inje University, Gimhae, Republic of Korea; ^2^Department of Medical Big Data, College of AI Convergence, Inje University, Gimhae, Republic of Korea

**Keywords:** depression, COVID-19 pandemic, CatBoost, machine learning, diabetic patients

## Abstract

**Objective:**

This study identified major risk factors for depression in community diabetic patients using machine learning techniques and developed predictive models for predicting the high-risk group for depression in diabetic patients based on multiple risk factors.

**Methods:**

This study analyzed 26,829 adults living in the community who were diagnosed with diabetes by a doctor. The prevalence of a depressive disorder was the dependent variable in this study. This study developed a model for predicting diabetic depression using multiple logistic regression, which corrected all confounding factors in order to identify the relationship (influence) of predictive factors for diabetic depression by entering the top nine variables with high importance, which were identified in CatBoost.

**Results:**

The prevalence of depression was 22.4% (*n* = 6,001). This study calculated the importance of factors related to depression in diabetic patients living in South Korean community using CatBoost to find that the top nine variables with high importance were gender, smoking status, changes in drinking before and after the COVID-19 pandemic, changes in smoking before and after the COVID-19 pandemic, subjective health, concern about economic loss due to the COVID-19 pandemic, changes in sleeping hours due to the COVID-19 pandemic, economic activity, and the number of people you can ask for help in a disaster situation such as COVID-19 infection.

**Conclusion:**

It is necessary to identify the high-risk group for diabetes and depression at an early stage, while considering multiple risk factors, and to seek a personalized psychological support system at the primary medical level, which can improve their mental health.

## 1. Introduction

Depression is the most common psychiatric disease in diabetic patients (1). Lustman et al. (1) conducted a large-scale epidemiological study on type 1 and type 2 diabetic patients and reported that the prevalence of depression in diabetic patients was approximately 30%, which was twice of that in non-diabetics. It could be a result of the physical, mental, and economic burden caused by diabetes and diabetic complications. However, the causal relationship between diabetes and depression has not yet been clearly understood, although approximately 30% of melancholic patients without diabetes are at risk of developing diabetes and depression is an independent risk factor for diabetes (2). Three mechanisms are suggested to explain why diabetes and depression are frequently accompanied: (1) stress due to increased intensity and repetition of diabetes treatment, (2) increased burden of other comorbidities and complications, and decreased quality of life due to the prolonged duration of diabetes, and (3) diabetes and depression share a common metabolic abnormality and are linked (3).

Depression has critically adverse effects on the prognosis of several chronic diseases (4). It especially leads to poor glycemic control by inducing diabetic patients to neglect self-care and reducing treatment compliance (4). Moreover, it eventually increases mortality by increasing the risk of microvascular complications and cardiovascular diseases (4). Since it has been reported that only 25% of diabetic patients are diagnosed with depression by medical personnel (5), early screening for depression is a very important issue for the patient’s prognosis and diabetes management.

Depression and diabetes are two conditions that have been significantly impacted by the COVID-19 pandemic. Both conditions can be influenced by lifestyle factors such as diet, exercise, and social support, and the disruptions caused by the pandemic have made it more challenging for individuals to manage these conditions. Especially since depression is affected by complex interactions among various factors, such as lifestyle and social networks rather than a single factor (6), it is necessary to develop a predictive model that considers multiple risk factors simultaneously in order to efficiently predict groups vulnerable to depression. Nevertheless, only a few studies have investigated multiple risk factors for depression in diabetic patients.

Many recent previous studies (6–8) used a Bayesian nomogram as a way to identify a high risk of disease by considering multiple risk factors. A nomogram is a graph that visualizes a prediction function derived from a Bayesian model or a logit model in two dimensions so that healthcare workers can easily interpret the derived results, and it is widely used in the healthcare field, such as predicting the risk of cancer recurrence (9). In particular, since the logistic nomogram has the advantage of being able to predict the probability of occurrence due to multiple risk factors by adding up individual risk factors (6), it can be effective for predicting depression in community diabetic patients after the COVID-19 pandemic. Therefore, this study identified major risk factors for depression in diabetic patients within the community using machine learning techniques and developed predictive models to identify the high-risk group for depression in diabetic patients based on multiple risk factors.

## 2. Materials and methods

### 2.1. Data source

It is an epidemiological study using the 2020 Community Health Survey data as secondary data. The Community Health Survey is conducted under the supervision of the Korea Disease Control and Prevention Agency to produce health statistics necessary for establishing a regional healthcare plan and implementing health projects. Please see Byeon (10) for a more detailed explanation of the data collection method and others of the Community Health Survey. Briefly explaining, the 2020 survey targeted adults (≥19 years old) based on resident registration in cities, counties, and districts nationwide and sampled using the systematic sampling method by extracting sampling points assigned to each region from the sampling frame created by linking the resident registration population data and housing data, which were complete enumeration data, and identifying the number of households selected as the sampling points. The survey was conducted from August 16th to October 31st, 2020, and a trained researcher conducted a 1:1 interview with the survey subject using a laptop computer (Computer Assisted Personal Interviewing, CAPI) to collect data. CAPI minimizes human errors and ensures accuracy through automated data collection and analysis. Additionally, CAPI enables fast and efficient data collection. The process of creating surveys and collecting data is automated, resulting in time and cost savings. This study analyzed 26,829 adults living in the community who were diagnosed with diabetes by a doctor in the 2020 Community Health Survey.

### 2.2. Measurement and definition of variables

The prevalence of a depressive disorder was the dependent variable in this study. The Korean version of the Patient Health Questionnaire (PHQ-9) (11) was used to assess depressive disorder. PHQ-9 is a standardized depression screening test developed by Spitzer et al. (12) to diagnose mental health in primary health care centers. It is made up of nine items that correspond to the Diagnostic and Statistical Manual of Mental Disorders (DSM-IV) diagnostic criteria for major depressive disorders. The PHQ-9 is a self-report test with high sensitivity and specificity (13). Furthermore, because it can simply check the severity of a depressive disorder using only nine items, it has the advantage of being highly likely to be applied to actual screening in epidemiological investigations as well as the medical field (13). The PHQ-9 asks a subject how frequently he or she has experienced anhedonia, depression, changes in sleep, fatigue, changes in appetite, guilt or worthlessness, decreased concentration, akathisia or feeling down, and suicidal thoughts in the previous 2 weeks. It is graded on a four-point scale: “never,” “for a few days,” “more than 1 week,” and “almost every day.” The total score ranges from 0 to 27, with a higher score indicating more severe depression. The threshold of depression was defined as 10 points (depression ≥ 10 points out of 27 points) based on the results of the previous studies (14, 15). Choi (16) reported that the sensitivity and specificity of PHQ-9 were 81.1 and 89.9%, respectively. Also, the reliability of the tool (Cronbach’s α) was 0.89 in this study. Based on the findings of previous studies (14, 15), the depression threshold defined as 10 points (depression ≥10 points out of a possible 27 points).

The explanatory variables included changes in instant food consumption before and after the COVID-19 pandemic (increased, similar, or decreased; responses were categorized based on self-report), changes in delivery food consumption before and after the COVID-19 pandemic (increased, similar, or decreased), changes in drinking before and after the COVID-19 pandemic (increased, similar, or decreased), changes in smoking before and after the COVID-19 pandemic (increased, similar, or decreased), changes in the use of public transportation before and after the COVID-19 pandemic (increased, similar, or decreased), satisfaction with life after the COVID-19 pandemic (satisfied or dissatisfied), age (40–49 years, 50–59 years, or ≥ 60 years), gender (male or female), residing location (urban or rural), education level (elementary school graduation or below, middle school graduation, high school graduation, college graduation or above), mean monthly household income [<South Korean won (KRW) 1.00 million, KRW 1.00 million – 2.99 million, KRW 3.00 million – 4.99 million, or ≥KRW 5.00 million], smoking (current smoker, former smoker, or non-smoker), subjective health (good, moderate, or poor), fear of infection due to the COVID-19 pandemic (yes, moderate, or no), fear of death due to the COVID-19 pandemic (yes, moderate, or no), concern about reproach from people around you due to the expression of COVID-19 symptoms (e.g., coughing) (yes, moderate, or no), concern about infection of health-vulnerable people such as infants and older adults among family members due to the COVID-19 pandemic (yes, moderate, or no), concern about economic loss due to the COVID-19 pandemic (yes, moderate, or no), changes in the number of meetings with people around you due to the COVID-19 pandemic (increased, similar, or decreased), changes in sleeping hours due to the COVID-19 pandemic (increased, similar, or decreased), marital status (living with a spouse or not living with a spouse), time of first diagnosed with diabetes (<60 years old or ≥60 years old), current non-drug treatment for diabetes (e.g., exercise) (yes or no), current diabetes drug (e.g., oral hypoglycemic drug) treatment (yes or no), current insulin injection treatment (yes or no), number of HbA1c tests in the past year (1 or fewer or 2 or more), diabetic eye disease complication test (fundus examination) in the past year (yes or no), diabetic renal complication test (microalbuminuria test) (yes or no), economic activity (yes or no), awareness of own blood glucose level (yes or no), awareness of own blood pressure (yes or no), number of days of conducting moderate-intensity (e.g., yoga and cycling) physical activity at least 30 min per day in the past week (none, 1–2 days, or 3 days or more), number of days of walking at least 30 min per day in the past week (none, 1–2 days, or 3 days or more), the number of people you can ask for help in a disaster situation such as COVID-19 infection (none, 1 ~ 2, or 3 or more), and diagnosis with hypertension (yes or no).

### 2.3. Development of a predictive model: categorical boosting

Categorical boosting (CatBoost) is a boosting algorithm that was developed in 2017 (17). It is designed to handle categorical variables efficiently and minimize model overfitting by using an ordered boosting technique. With CatBoost, categorical variables can be used without the need to convert them into numbers. The algorithm also automatically applies a suitable encoding technique for categorical variables, such as one-hot encoding, target encoding, mean encoding, and response encoding (17). Additionally, CatBoost optimizes hyperparameters with an internal algorithm instead of using special hyperparameter optimization, making it easier to use compared to other algorithms that require hyperparameter tuning. This study set the regularization lambda, the number of trees, the limit depth of individual trees to 6, and the learning rate of CatBoost to 3, 100, 6, and 0.300, respectively. This study calculated the importance of variables based on the mean decrease in impurity and selected the top 9 variables with high importance.

### 2.4. Development and verification of logistic monogram

When the number of risks included in the nomogram increases, the number of cases needed to calculate the predicted probability also increases. This study developed a model for predicting diabetic depression using multiple logistic regression, which corrected all confounding factors in order to identify the relationship (influence) of predictive factors for diabetic depression by entering the top nine variables with high importance, which were identified in CatBoost. This study used an adjusted odds ratio (aOR) and 95% confidence interval (CI) to identify the independent relationship between predictors and diabetic depression.

The developed model for predicting depression in individuals with diabetes presented a graph by establishing a nomogram, which allows healthcare workers to easily interpret the probability of high-risk groups based on multiple risk factors. The nomogram developed in this study consisted of four lines. Firstly, the point line was placed at the top of the nomogram to derive scores corresponding to the categories of risk factors, and the point line of the Bayesian nomogram was between 0 and 100 points. Secondly, there were as many risk factor lines as the number of risk factors. Thirdly, the total point line was the sum of each individual risk factor and was located at the bottom of the nomogram. Finally, the probability line was placed at the bottom of the nomogram to derive the probability of depression in individuals with diabetes.

The prediction performance of the finally developed diabetic depression prediction nomogram was evaluated using the 10-fold cross-validation method. This study used F1-score, the area under the curve (AUC), general accuracy, precision, recall, and calibration plot as evaluation indices to confirm the predictive performance.

## 3. Results

### 3.1. General characteristics according to the depression prevalence in diabetic patients after the COVID-19 pandemic

Table 1 shows the characteristics of the subjects according to the depression prevalence in diabetic patients in South Korea. Among 26,829 diabetic patients, the prevalence of depression was 22.4% (*n* = 6,001). The results of chi-square test revealed that diabetic depression was significantly affected by changes in instant food consumption before and after the COVID-19 pandemic, changes in delivery food consumption before and after the COVID-19 pandemic, changes in drinking before and after the COVID-19 pandemic, changes in smoking before and after the COVID-19 pandemic, satisfaction with life after the COVID-19 pandemic, gender, residing location, education level, mean monthly household income, smoking, subjective health, fear of infection due to the COVID-19 pandemic, fear of death due to the COVID-19 pandemic, concern about reproach from people around you due to the expression of COVID-19 symptoms, concern about infection of health-vulnerable people due to the COVID-19 pandemic, concern about economic loss due to the COVID-19 pandemic, changes in sleeping hours due to the COVID-19 pandemic, marital status, time of first diagnosed with diabetes, current insulin injection treatment, diabetic eye disease complication test in the past year, diabetic renal complication test, economic activity, awareness of own blood glucose level, awareness of own blood pressure, number of days of conducting moderate-intensity physical activity at least 30 min per day in the past week, number of days of walking at least 30 min per day in the past week, the number of people you can ask for help in a disaster situation such as COVID-19 infection, and diagnosis with hypertension (*p* < 0.05).

**Table 1:** General characteristics according to depression prevalence in diabetic patients after the COVID-19 pandemic, *n* (%).

Variable	Depression		***p***
	Yes (*n* = 6,001)	No (*n* = 20,828)	
Age			0.262
40–49 years	471 (23.6)	1,527 (76.4)	
50–59 years	1,050 (22.7)	3,571 (77.3)	
≥60 years	4,448 (22.1)	15,656 (77.9)	
Gender			<0.001
Male	2,289 (17.3)	10,926 (82.7)	
Female	3,712 (27.3)	9,902 (72.7)	
Residing location			<0.001
Urban	3,151 (24.8)	9,565 (75.2)	
Rural	2,850 (20.2)	11,263 (79.8)	
Education level			<0.001
Elementary school graduation or below	2,722 (25.3)	8,020 (74.7)	
Middle school graduation	1,077 (22.8)	3,647 (77.2)	
High school graduation	1,427 (20.2)	5,721 (80.0)	
College graduation or above	765 (18.3)	3,415 (81.7)	
Mean monthly household income			<0.001
<KRW 1.00 million	1,923 (29.7)	4,555 (70.3)	
KRW 1.00 million – 2.99 million	1,938 (22.2)	6,811 (77.8)	
KRW 3.00 million – 4.99 million	760 (19.3)	3,181 (80.7)	
≥KRW 5.00 million	533 (17.3)	2,551 (82.7)	
Smoking			<0.001
Current smoker	961 (22.9)	3,238 (77.1)	
Former smoker	1,177 (17.5)	5,538 (82.5)	
Non-smoker	3,863 (24.3)	12,049 (75.7)	
Subjective health			<0.001
Good	735 (10.9)	6,009 (89.1)	
Moderate	2,124 (18.3)	9,482 (81.7)	
Poor	3,141 (37.1)	5,336 (62.9)	
Changes in instant food consumption before and after the COVID-19 pandemic			<0.001
Increased	332 (29.6)	788 (70.4)	
Similar	1,935 (21.0)	7,291 (79.0)	
Decreased	571 (24.4)	1,768 (75.6)	
Changes in delivery food consumption before and after the COVID-19 pandemic			<0.001
Increased	484 (25.9)	1,383 (74.1)	
Similar	1,188 (20.5)	4,596 (79.5)	
Decreased	419 (24.2)	1,309 (75.8)	
Changes in drinking before and after the COVID-19 pandemic			<0.001
Increased	148 (31.2)	327 (68.8)	
Similar	1,100 (19.0)	4,700 (81.0)	
Decreased	950 (20.4)	3,705 (79.6)	
Changes in smoking before and after the COVID-19 pandemic			<0.001
Increased	149 (35.6)	269 (64.4)	
Similar	804 (20.1)	3,194 (79.9)	
Decreased	307 (20.9)	1,161 (79.1)	
Changes in the use of public transportation before and after the COVID-19 pandemic			0.155
Increased	47 (30.7)	106 (69.3)	
Similar	1,011 (24.4)	3,126 (75.6)	
Decreased	2,484 (25.3)	7,336 (74.7)	
Satisfaction with life after the COVID-19 pandemic			<0.001
Dissatisfied	1,314 (27.2)	3,523 (72.8)	
Satisfied	4,630 (21.3)	17,103 (78.7)	
Fear of infection due to the COVID-19 pandemic			<0.001
Yes	4,653 (23.5)	15,166 (76.5)	
Moderate	810 (19.6)	3,323 (80.4)	
No	529 (18.5)	2,333 (81.5)	
Fear of death due to the COVID-19 pandemic			<0.001
Yes	3,660 (24.2)	11,459 (75.8)	
Moderate	1,051 (20.8)	3,994 (79.2)	
No	1,279 (19.3)	5,352 (80.7)	
Concern about reproach from people around you due to the expression of COVID-19 symptoms			<0.001
Yes	4,814 (22.9)	16,229 (77.1)	
Moderate	617 (21.0)	2,318 (79.0)	
No	553 (19.7)	2,250 (80.3)	
Concern about infection of health-vulnerable people such as infants and older adults among family members due to the COVID-19 pandemic			<0.001
Yes	5,065 (23.0)	16,954 (77.0)	
Moderate	326 (18.7)	1,416 (81.3)	
No	218 (18.0)	993 (82.0)	
Concern about economic loss due to the COVID-19 pandemic			<0.001
Yes	5,083 (23.1)	16,940 (76.9)	
Moderate	483 (20.1)	1,918 (79.9)	
No	423 (17.7)	1,964 (82.3)	
Changes in the number of meetings with people around you due to the COVID-19 pandemic			0.578
Increased	23 (25.8)	66 (74.2)	
Similar	797 (21.4)	2,931 (78.6)	
Decreased	4,545 (21.7)	16,419 (78.3)	
Changes in sleeping hours due to the COVID-19 pandemic			<0.001
Increased	626 (26.3)	1,753 (73.7)	
Similar	4,598 (20.6)	17,754 (79.4)	
Decreased	776 (37.0)	1,320 (63.0)	
Marital status			<0.001
Living with a spouse	3,449 (19.2)	14,539 (80.8)	
Not living with a spouse	2,552 (28.9)	6,289 (71.1)	
Time of first diagnosis with diabetes			0.010
<60 years old	3,394 (22.9)	11,398 (77.1)	
≥60 years old	2,588 (21.6)	9,375 (78.4)	
Current non-drug treatment for diabetes (e.g., exercise)			0.241
Yes	2,160 (22.0)	7,671 (78.0)	
No	3,839 (22.6)	13,155(77.4)	
Current diabetes drug (e.g., oral hypoglycemic drug) treatment			0.239
Yes	5,468 (22.3)	19,080 (77.7)	
No	531 (23.4)	1,743 (76.6)	
Current insulin injection treatment			<0.001
Yes	596 (30.4)	1,367 (69.6)	
No	5,400 (21.7)	19,457 (78.3)	
Number of HbA1c tests in the past year			0.078
1 or less	2,768 (22.8)	9,357 (77.2)	
2 or more	3,195 (21.9)	11,376 (78.1)	
Diabetic eye disease complication test (fundus examination) in the past year			<0.001
Yes	2,614 (23.7)	8,425 (76.3)	
No	3,358 (21.4)	12,343 (78.6)	
Diabetic renal complication test (microalbuminuria test)			0.040
Yes	2,929 (22.9)	9,880 (77.1)	
No	3,017 (21.8)	10,812 (78.2)	
Economic activity			<0.001
Yes	2,159 (16.6)	10,844 (83.4)	
No	3,841 (27.8)	9,977 (72.2)	
Awareness of own blood glucose level			<0.001
Yes	4,444 (21.6)	16,140 (78.4)	
No	1,539 (24.8)	4,658 (75.2)	
Awareness of own blood pressure			<0.001
Yes	4,260 (21.4)	15,607 (78.6)	
No	1,723 (24.9)	5,186 (75.1)	
Number of days of conducting moderate-intensity (e.g., yoga and cycling) physical activity at least 30 min per day in the past week			<0.001
None	4,679 (23.4)	15,276 (76.6)	
1–2 days	381 (20.6)	1,465 (79.4)	
3 days or more	937 (18.7)	4,068 (81.3)	
Number of days of walking at least 30 min per day in the past week			<0.001
None	1,928 (25.8)	5,553 (74.2)	
1–2 days	670 (24.5)	2,069 (75.5)	
3 days or more	3,402 (20.5)	13,198 (79.5)	
Number of people you can ask for help in a disaster situation such as COVID-19 infection			<0.001
None	1,724 (27.6)	4,517 (72.4)	
1–2 people	2,637 (23.0)	8,817 (77.0)	
3 people or more	1,633 (18.0)	7,453 (82.0)	
Diagnosis with hypertension			<0.001
Yes	3,910 (23.6)	12,648 (76.4)	
No	2,090 (20.4)	8,177 (79.6)	

### 3.2. Predictors of depression in diabetic patients living in South Korean community

This study calculated the importance of factors related to depression in diabetic patients living in South Korean community using CatBoost to find that the top nine variables with high importance were gender, smoking status, changes in drinking before and after the COVID-19 pandemic, changes in smoking before and after the COVID-19 pandemic, subjective health, concern about economic loss due to the COVID-19 pandemic, changes in sleeping hours due to the COVID-19 pandemic, economic activity, and the number of people you can ask for help in a disaster situation such as COVID-19 infection.

Table 2 presents the results of logistic regression analysis for predicting depression in diabetic patients living in the South Korean community the top nine variables with high impact on model output in CatBoost. The analysis results of the adjusted model for predicting depression in South Korean diabetic patients showed that female (AOR = 1.78, 95% CI = 1.68, 1.89), current smoker (AOR = 1.39, 95% CI = 1.26, 1.53), concern about economic loss due to the COVID-19 pandemic (moderate: AOR = 1.19; yes: AOR = 1.39), changes in sleeping hours due to the COVID-19 pandemic (similar: AOR = 0.60; decreased: AOR = 1.38), changes in drinking before and after the COVID-19 pandemic (similar: AOR = 1.76; increased: AOR = 1.93), changes in smoking before and after the COVID-19 pandemic (similar: AOR = 2.09; increased: AOR = 2.20), subjective health (moderate: AOR = 2.62; poor: AOR = 4.81), and the number of people you can ask for help in a disaster situation such as COVID-19 infection (1–2 persons: AOR = 1.27; none: AOR = 1.74) were independent factors of depression in diabetic patients (*p* < 0.05).

**Table 2:** Predictors of depression in diabetic patients living in the South Korean community: AOR and 95% CI.

Variables	AOR	95%CI	*p*
Changes in drinking before and after the COVID-19 pandemic			
Increased	1.93	1.57, 2.37	<0.001
Similar	1.76	1.43, 2.17	<0.001
Decreased (ref)	1	1	
Changes in smoking before and after the COVID-19 pandemic			
Increased	2.20	1.77, 2.72	<0.001
Similar	2.09	1.65, 2.65	<0.001
Decreased (ref)	1	1	
Gender			
Male (ref)	1	1	
Female	1.78	1.68, 1.89	<0.001
Smoking			
Current smoker	1.39	1.26, 1.53	<0.001
Former smoker	0.92	0.85, 1.00	0.060
Non-smoker (ref)	1	1	
Subjective health			
Good (ref)	1	1	
Moderate	2.62	2.46, 2.80	<0.001
Poor	4.81	4.40, 5.25	<0.001
Concern about economic loss due to the COVID-19 pandemic			
Yes	1.39	1.24, 1.55	0.001
Moderate	1.19	1.07, 1.32	<0.001
No (ref)	1	1	
Changes in sleeping hours due to the COVID-19 pandemic			
Increased (ref)	1	1	
Similar	0.60	0.53, 0.69	<0.001
Decreased	1.38	1.25, 1.51	<0.001
Economic activity			
Yes (ref)	1	1	
No	1.93	1.82, 2.05	<0.001
Number of people you can ask for help in a disaster situation such as COVID-19 infection			
None	1.74	1.61, 1.88	0.001
1–2 people	1.27	1.18, 1.36	0.001
3 people or more (ref)	1	1	

### 3.3. Development and validation of depression predictive nomogram for diabetic patients living in the South Korean community

**Figure 1:**
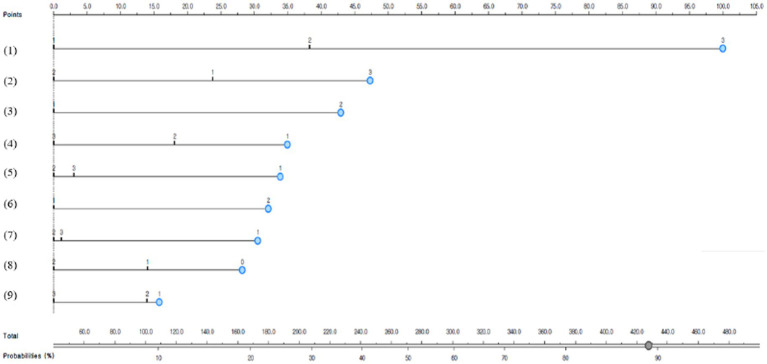
Depression predictive nomogram for South Korean diabetic patients; (1) Subjective health: 1, good; 2, moderate; or 3, poor; (2) changes in sleeping hours after the COVID-19 pandemic: 1, increased; 2, similar; or 3, decreased; (3) Gender: 1, male, or 2, female; (4) smoking status: 1, current smoker; 2, former smoker; or 3, non-smoker; (5) changes in drinking after the COVID-19 pandemic: 1, increased; 2, similar; 3, decreased; (6) economic activity: 1, yes; or 2, no; (7) changes in smoking after the COVID-19 pandemic: 1, increased; 2, similar; or 3, decreased; (8) people whom you can ask for help: 0, 0 people; 1, 1–2 people; or 2, 3 or more people; and (9) concern about economic loss due to the COVID-19 pandemic: 1, yes; 2, moderate; or 3, no.

Figure 1 presents the depression predictive nomogram for diabetic patients living in the South Korean community. The nomograph (Figure 1) analyzed the high-risk group for depression in diabetic patients and predicted that female diabetic patients who had fewer sleeping hours after the COVID-19 pandemic, increased the frequency of smoking and drinking increased than before the pandemic, concerned about economic loss due to the COVID-19 pandemic, had no one to ask for help, and perceived subjective health as poor had an 88% predictive possibility of depression.

**Figure 2:**
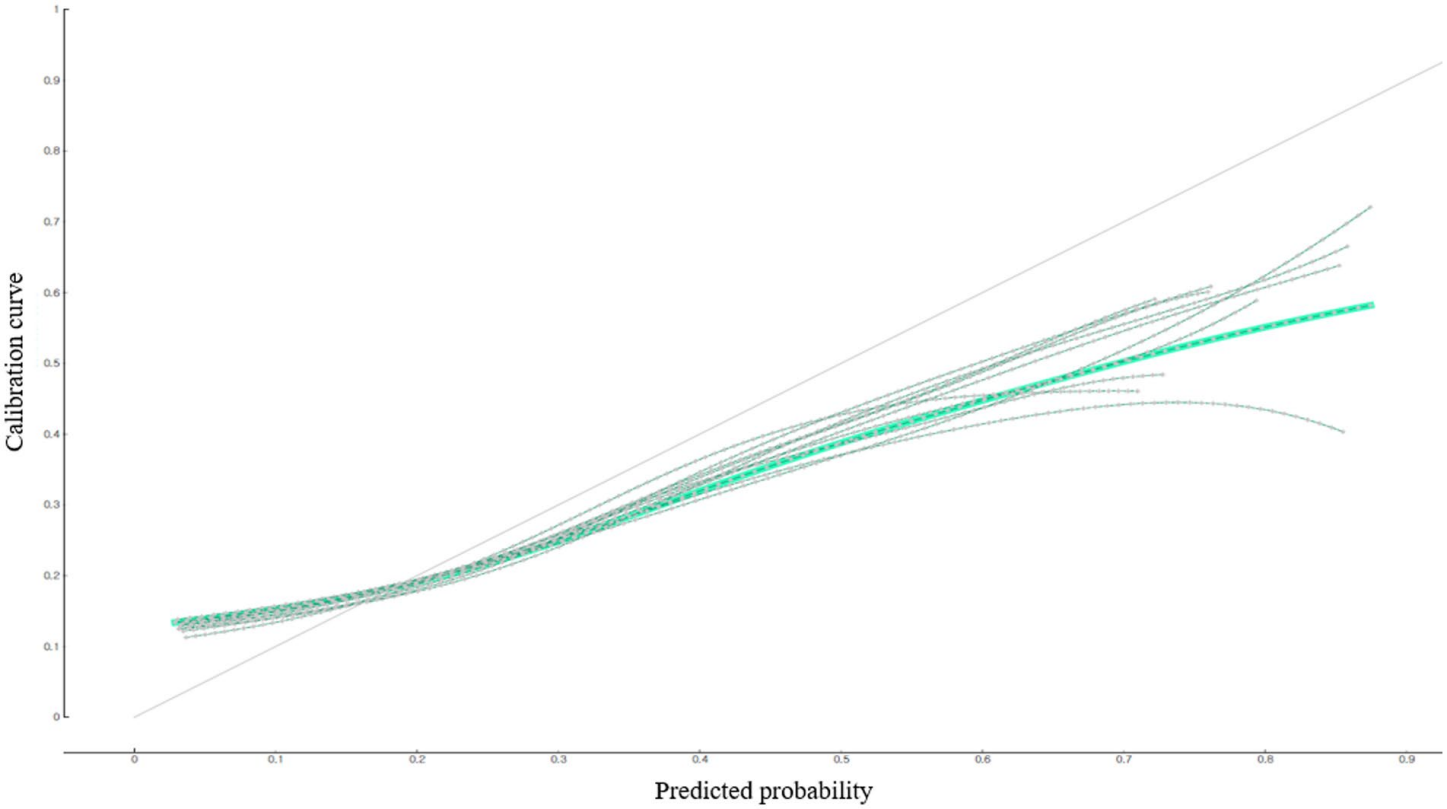
Predictive performance of the depression predictive nomogram for South Korean diabetic patients: calibration plot.

**Figure 3:**
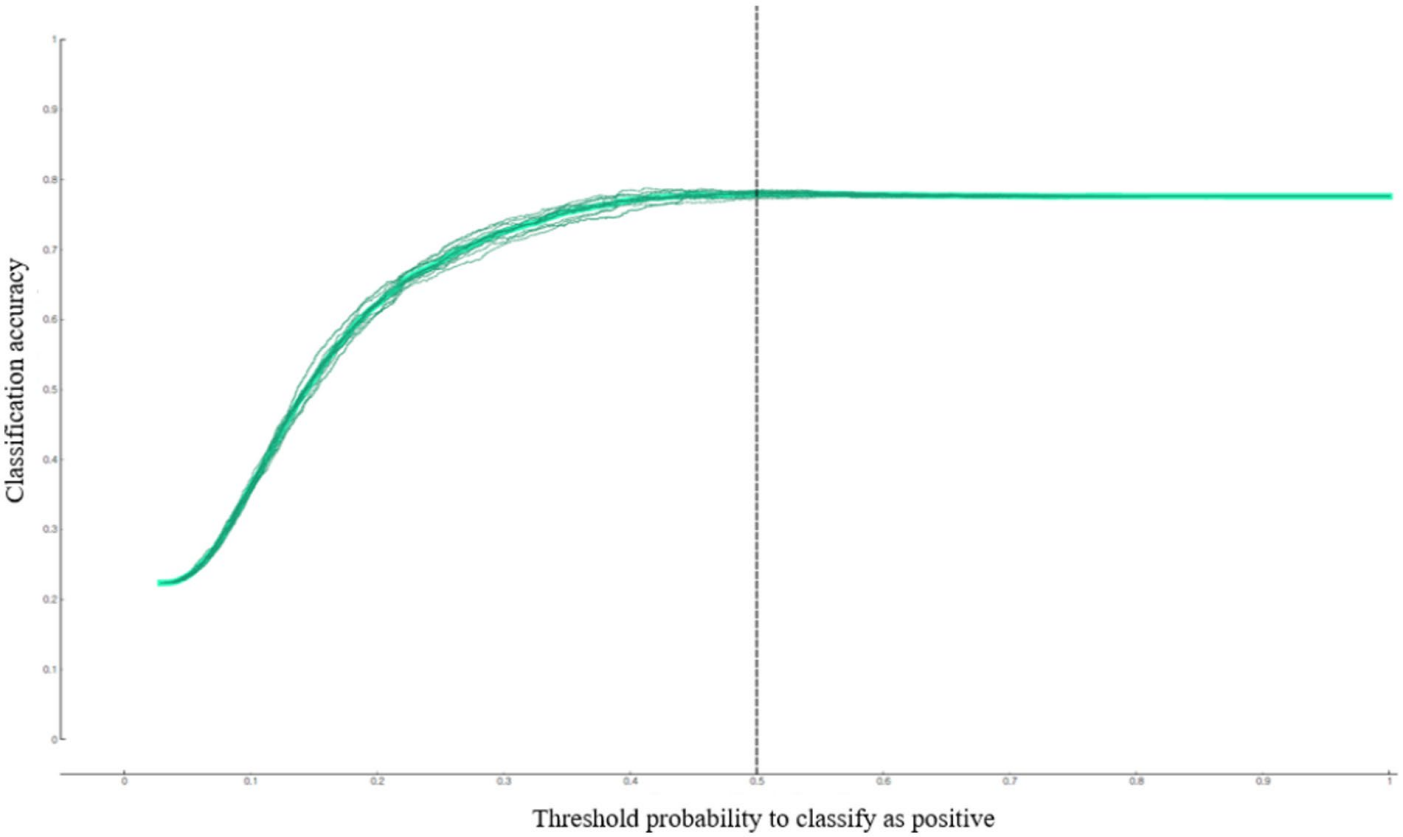
Predictive performance of the depression predictive nomogram for South Korean diabetic patients: accuracy.

This study examined the predictive performance of the developed depression predictive nomogram for diabetic patients living in South Korea using calibration plot (Figure 2), AUC, and accuracy (Figure 3). This study compared the prediction probability and observation probability of the diabetic patient group with depression with those of the diabetic patient group without depression using calibration plot and chi-square test (Figure 2). The prediction probability and observation probability were not significantly different (*p* < 0.05). The results of 10-fold cross validation showed that AUC, general accuracy, precision, recall, and F1-score were 0.704, 0.780, 0.735, 0.780, and 0.712, respectively.

## 4. Discussion

This study identified the prevalence of depression among diabetic patients living in South Korean local communities using national survey data conducted after the COVID-19 pandemic and found that 22.4% of the subjects were diabetic patients with depression. The prevalence of depression among diabetic patients living in South Korean local communities was approximately twice the prevalence of depression among healthy people (12%) during the period (18). Although it cannot be directly compared with the results of this study, the meta-analysis of Anderson et al. (19), conducted before the COVID-19 pandemic, reported that depression in diabetic patients (28.5%) was 1.5 times higher than that in the general population (16.2%). Even though diabetic patients in local communities are at high risk of depression, not enough active attention has been given to their emotional aspects. It has been reported that when depression accompanies diabetes, medical costs increase because glycemic control deteriorates, the incidence of chronic complications increases, and mortality rises (20–22). Furthermore, if a diabetic patient cannot properly perform health behaviors due to depression, it can adversely affect the long-term course of diabetes, such as the occurrence of chronic complications, as well as glycemic control (23). Consequently, in order to efficiently screen depression in diabetic patients at an early stage, studies need to identify the risk factors for depression.

The results of this study confirmed that gender, subjective health, increased health risk behaviors such as drinking and smoking, decreased sleeping hours, and the number of people whom you could seek help in a disaster situation such as COVID-19 infection were independent risk factors of depression. These results agreed with the results of previous studies (24–27). Female sex, marital status, childhood adversity, and social deprivation are general population risk factors for depression that also apply to people with diabetes (28).

Gender is known to be a major factor influencing diabetic patients. Adriaanse et al. (24) analyzed depressive symptoms in type 2 diabetic patients and reported that the prevalence of depression was significantly higher in women (15%) than in men (9.1%), which concurred with the results of this study. Moreover, a decrease in sleeping hours has been reported as a significant predictor of diabetic depression. Ghosh et al. (25) showed that a quarter of diabetic patients with depression experienced a decrease in sleeping hours. Particularly, the number of people subjects could ask for help in a disaster situation was a key factor related to diabetic depression. Social support is known to be another major risk factor for diabetic depression. Pibernik-Okanovic (26) found that diabetic patients who felt a lack of social support had a higher risk of developing depression, which supported the results of this study. Therefore, it is necessary to develop a psychological support program for diabetic patients in the community to increase their ability to cope with depression caused by social distancing in the era of COVID-19 and limited social contact and build a system that can continuously provide medical and social support for diabetic patients without sufficient social support to prevent depression in diabetic patients.

In this study, the number of diabetic complication tests in the past year, awareness of blood glucose levels, and diabetic treatment methods (e.g., oral hypoglycemic drug and insulin injection treatment) were not related to depression in diabetic patients, which did not agree with the results of previous studies (1, 29, 30). Numerous studies (1, 29) have proven the relationship between glycemic control and depression in diabetic patients. When glycemic control was poorer, depression symptoms were more severe (29). It is backed by the results that a higher level of glycated hemoglobin decreases the effects of antidepressants (1). It is speculated that the awareness of blood glucose levels or the number of diabetes complication tests based on a survey alone was not enough to directly identify the blood glucose management level of diabetic patients. Therefore, although previous studies (1, 29) reported that managing blood glucose is related to the depression of diabetic patients, the relationship was not significant in this study.

The mechanism underlying the high risk of depression in diabetic patients has not been clearly understood. There are some possible explanations: depression may induce insulin resistance by stimulating the secretion of insulin-antagonizing hormones (e.g., catecholamines, glucocorticoids, growth hormones, and glucagon) and inflammatory cytokines, or it can contribute to the development of diabetes by causing dysfunction of pancreatic beta cells. Moreover, diabetes may cause depression in association with inadequate glycemic control, the development of chronic complications due to diabetes, and a decline in socioeconomic status. However, since depression is caused by multiple factors rather than a single factor, future studies need to identify the relationship between blood glucose management and depression using clinical test data such as HbA1c level in addition to sociodemographic and psychological characteristics.

Another finding of this study was that the results of this study showed that “female diabetic patients who had fewer sleeping hours after the COVID-19 pandemic, increased the frequency of smoking and drinking increased than before the pandemic, concerned about economic loss due to the COVID-19 pandemic, had no one to ask for help, and perceived subjective health as poor had an 88% predictive possibility of depression,” which was high. Since multiple risk factors for diabetic depression have not been clearly identified, it is needed to carry out future studies on multiple risk factors for diabetic depression based on large-scale cohort data. It is also necessary to continuously monitor depression in terms of primary care for diabetic patients with these multiple risk factors.

In the United States, the Centers for Disease Control and Prevention regularly conducts regular monitoring of comorbidities in diabetic patients and runs a chronic disease prevention program (31). On the other hand, South Korea lacks a systematic monitoring system for diabetic depression management, and previous studies mainly examined multi-center registry data for diabetic depression (32, 33). Particularly, in South Korea, mental health management and education such as depression for diabetic patients are mainly carried out in general hospitals (32). Considering the fact that general hospitals are playing a critical role in the emergency medical response system in a disaster situation such as the COVID-19 pandemic (34), it will be required to establish a systematic depression examination and monitoring system centered on primary care in the future for the sustainability mental health management of diabetic patients.

The strength of this study was that it identified a high-risk group for depression in diabetic patients using national survey data conducted after the COVID-19 pandemic and provided baseline data for preventing depression in diabetic patients. This study had several limitations. First, since this study analyzed secondary data by analyzing epidemiological data (survey data), clinical indicators such as insulin-antagonizing hormones and genes related to depression were not included. Second, the in-person survey may underestimate health risk behaviors such as smoking and drinking. Therefore, future studies need to reduce the possibility of recall bias including medical records in order to identify factors related to diabetic depression. Third, the Community Health Survey, the source data, did not survey the duration of diabetes. Future studies need to investigate the duration of diabetes and duration of diabetic complications additionally to develop a diabetes depression predictive model with higher predictive performance. Fourth, since this study was a cross-sectional study, even if risk factors for diabetic depression were identified in this study, it could not be interpreted as a causal relationship based on temporal precedence.

## 5. Conclusion

It is necessary to identify the high-risk groups for diabetes and depression at an early stage while considering multiple risk factors and provide a tailored psychological support system at the primary medical level to improve their mental health. Additionally, it is important to establish a system that can systematically monitor the high-risk groups for diabetes and depression, even in long-term disaster situations (e.g., pandemic), at the community level. Furthermore, additional longitudinal studies are needed to confirm the causal relationship between factors related to diabetic depression identified in this study.

## Data availability statement

The datasets presented in this study can be found in online repositories. The names of the repository/repositories and accession number(s) can be found at: https://chs.kdca.go.kr/chs/index.do.

## Ethics statement

The studies involving human participants were reviewed and approved by the Institutional Review Board (or Ethics Committee) of Korea Disease Control and Prevention Agency (protocol code: 117075 and date: 2021.07.01) and the study was conducted according to the guidelines of the Declaration of Helsinki. The patients/participants provided their written informed consent to participate in this study.

## Author contributions

HB designed the manuscript, was involved in study data interpretation, preformed the statistical analysis, and assisted with writing the manuscript.

## Funding

This research was supported by Basic Science Research Program through the National Research Foundation of Korea (NRF) funded by the Ministry of Education (NRF-2018R1D1A1B07041091 and 2021S1A5A8062526).

## Conflict of interest

The author declares that the research was conducted in the absence of any commercial or financial relationships that could be construed as a potential conflict of interest.

## Publisher’s note

All claims expressed in this article are solely those of the authors and do not necessarily represent those of their affiliated organizations, or those of the publisher, the editors and the reviewers. Any product that may be evaluated in this article, or claim that may be made by its manufacturer, is not guaranteed or endorsed by the publisher.
